# Access to automated comparative feedback reports in primary care – a study of intensity of use and relationship with clinical performance among Swedish primary care practices

**DOI:** 10.1186/s12913-023-10407-9

**Published:** 2024-01-04

**Authors:** Anders Anell, Eva Arvidsson, Margareta Dackehag, Lina Maria Ellegård, Anna Häger Glenngård

**Affiliations:** 1 Lund University School of Economics & Management, Lund, Sweden; 2 Futurum, Region Jönköping County, Jönköping, Sweden; 3 School of Health and Welfare, Jönköping University, Jönköping, Sweden; 4 Faculty of Business, Kristianstad University, Kristianstad, Sweden

**Keywords:** Primary health care, General practice, Quality improvement, Automated feedback reports, Audit and feedback, Quality indicators, Electronic medical records, Motivation

## Abstract

**Background:**

Digital applications that automatically extract information from electronic medical records and provide comparative visualizations of the data in the form of quality indicators to primary care practices may facilitate local quality improvement (QI). A necessary condition for such QI to work is that practices actively access the data. The purpose of this study was to explore the use of an application that visualizes quality indicators in Swedish primary care, developed by a profession-led QI initiative (“Primärvårdskvalitet”). We also describe the characteristics of practices that used the application more or less extensively, and the relationships between the intensity of use and changes in selected performance indicators.

**Methods:**

We studied longitudinal data on 122 primary care practices’ visits to pages (page views) in the application over a period up to 5 years. We compared high and low users, classified by the average number of monthly page views, with respect to practice and patient characteristics as well as baseline measurements of a subset of the performance indicators. We estimated linear associations between visits to pages with diabetes-related indicators and the change in measurements of selected diabetes indicators over 1.5 years.

**Results:**

Less than half of all practices accessed the data in a given month, although most practices accessed the data during at least one third of the observed months. High and low users were similar in terms of most studied characteristics. We found statistically significant positive associations between use of the diabetes indicators and changes in measurements of three diabetes indicators.

**Conclusions:**

Although most practices in this study indicated an interest in the automated feedback reports, the intensity of use can be described as varying and on average limited. The positive associations between the use and changes in performance suggest that policymakers should increase their support of practices’ QI efforts. Such support may include providing a formalized structure for peer group discussions of data, facilitating both understanding of the data and possible action points to improve performance, while maintaining a profession-led use of applications.

**Supplementary Information:**

The online version contains supplementary material available at 10.1186/s12913-023-10407-9.

## Background

Clinical audit and feedback interventions have attracted considerable scholarly interest, and a number of studies have assessed the relationship between the design of interventions and impact on professionals’ behavior. Previous research [1–3] emphasizes that data should be validated and up-to-date, and provided by a trusted and legitimate source (supervisor or colleague) using multimodal forms (i.e. using both text-based feedback, visualization aids and face-to-face meetings). Moreover, feedback should include comparisons with other relevant practices and support the development of local action plans. The development of digital applications that automatically extract information from electronic medical records (EMR) promises many benefits. Visualizations of up-to-date data extracted from EMRs, including comparisons with other providers based on evidence-based indications and targets, may support clinical decisions at the point of care, facilitate quality improvement (QI) initiatives at the provider level and contribute to clinical research [4].

Having access to valid and up-to-date data is only a first necessary step in QI initiatives, however. To affect clinical outcomes, professionals also need to actively use the data, and, informed by the data, adjust their clinical behavior [5]. The eventual impact of improving access to data and comparisons is likely to depend on several contextual factors, such as recipient´s capabilities and motivation, and the degree to which the existing organizational, financial and regulatory conditions provide opportunities for change [1–3, 6]. A systematic review [1] suggests a more visible impact among providers with poor performance and in situations where the data prompts simple rather than complex changes, i.e., if recipients of feedback are able to implement changes individually rather than collectively.

A recent review on drives for QI in primary care concludes that more evidence on non-financial incentives for QI in primary care is needed [7]. This study reports findings from a study of a digital application supporting automated feedback reports in Swedish primary care (PC), specifically intended to support QI initiatives led by professionals. As PC both in Sweden and in other countries is expected to take the main responsibility for first-line care, chronic care, and the coordination of care performed by others [8–10], such QI initiatives are much needed. At the same time, PC in many countries face staff and resource shortages in parallel to increasing workloads, leading to elevated levels of stress and dissatisfaction with the work environment [11–13]. The shortage of GPs limits the time available for QI initiatives that potentially could improve PC performance and ease the burden on GPs, making access to automated reports even more important. Another factor that has constrained QI work is the scarcity of updated and relevant data on clinical performance. Also in this respect, automated reports may create opportunities for more active use of data. Given the volume and breadth of conditions handled in PC, the need for automated tools to aggregate and make sense of the data may also be larger in PC compared to other medical specialities [13].

*Primary Care Quality* (PCQ) is a recent Swedish initiative aimed to facilitate profession-led quality improvement work at PC practices. PCQ was initiated by leading PC professionals and is still run by a group of GPs, nurses, physiotherapists, occupational therapists and psychologists working in primary care. It is financed collectively by the Swedish regional health authorities. PCQ consists of a library of 150 + quality indicators for acute and chronic conditions, mental illness, rehabilitation, and core areas such as continuity of care, multimorbidity and lifestyle habits (see Additional file 1). The indicators are developed by the group of PC professionals and are based on evidence and national guidelines. Each indicator definition includes a detailed description of the relevant diagnoses, time period, type of contact, type of provider, laboratory results etc. An external digital application that retrieves the data from the EMR is needed to visualize the PCQ indicators.

The initiative has a strong emphasis on being a profession-led, local QI tool, rather than a traditional audit and feedback intervention focusing accountability towards targets. Therefore, reports based on PCQ indicators are not automatically disseminated to care providers; users need to log in to a data visualization application to see their data. The data used to construct the indicators is automatically obtained from EMRs and visualized in diagrams and tables which allow the intended users – practice managers and medical professionals – to compare the performance of their own practice to that of other practices in Sweden [14]. Via the application, PC professionals can also identify patients who may need attention. Thus, PCQ facilitates the analysis, reflection and learning based on follow-up and comparison of data.

The PCQ group have regular meetings with users all over Sweden to evaluate and, if needed, revise the indicators, but it is not known to what extent the PCQ indicators are actually used to support QI in primary care practices. The purpose of this study was to study the early diffusion and use of PCQ indicators among PC practices in one large Swedish region. In addition, we aimed to characterize care providers that used the data relatively intensively (in terms of page views of PCQ indicators), and to describe relationships between the intensity of use and changes in selected performance indicators over time.

## Setting

The Swedish healthcare system is financed and organized by 21 regional health authorities. Each region stipulates the requirements (financial, organizational and quality standards) for the publicly funded PC practices (PCP). The same requirements apply to both public and private PCPs [15]. Payment to PCPs mainly consists of risk-adjusted capitation. PCPs have a comprehensive financial responsibility for providing primary care to registered patients, including prescribed medicines.

PCPs typically have 40–50 employees, including general practitioners (GP), nurses with different specializations, and physiotherapists [16, 17]. The team-based multi-professional PCP structure facilitates a flexible use of resources, e.g. task shifting between physicians and nurses. Each PCP has a managing director in charge of operations. In case the managing director is not a GP by profession, there must also be an appointed clinical director, a GP, with the overall medical responsibility.

The first version of the PCQ library was made available in 2016. To date, around 95% of PCPs have access to applications that automatically extract data from EMRs and visualize the data according to the PCQ indicator descriptions. The data for this study was collected from a large Swedish region with 1.8 million inhabitants and 200 private and public PCPs. In 2016, the region started a pilot in which some PCPs agreed to provide data, and thus get access to PCQ indicators for their practice. The PCPs in the pilot generally found the data visualizations useful, but also suggested ways to improve the application, e.g., to add a possibility to group the results by age and gender and to clarify what data was used in the numerator and denominator of each indicator (these suggestions were implemented). In 2017, the region offered all PCPs in the region the opportunity to connect their EMRs to the application, still on a voluntary basis. The region decided to connect all publicly owned PCPs to the application from the fall of 2018. Since 2019, it is mandatory for all publicly funded PCPs (including private PCPs with public funding) in the region to enable the functionality.

The study region visualizes the PCQ data using an application provided by a private company, Medrave Software. Since the application (henceforth denoted “MR”) also has other functionalities, users – who may be either managers or professionals – need to click their way through a menu system to access the PCQ section of MR. The index page of the PCQ section displays boxplots of the national distributions of several summary indicators, sorted in overarching areas (chronic conditions, infections etc.). It is easy for users to get an overview of how the measurement for their PCP compares to other practices in the country or region. From the index page, the user may select to drill down into specific areas. For instance, a user interested in diabetes care can by one click reach the subsection showing diagrams of PCQ indicators related to diabetes. It is possible to filter indicators by type (measurements of, e.g., HbA1c, type of diabetes treatment, follow-up visits), and to click on specific indicator names to get more information on performance over time. The user can also access list of the relevant patients for each indicator, e.g., the patients with diabetes who had not had a follow-up visit recently.

Apart from the PCQ section, the MR application has a so-called report generator, which can be used to extract information. For instance, users may generate reports showing all patients at the practice with a certain diagnosis or prescribed medicine, or reports showing statistics about visits, lab tests or referrals or issued sickness certificates. It is also possible to generate reports for certain common diseases such as asthma, COPD, diabetes, hypertension, and tonsillitis. The disease-specific MR reports include some measures that are similar to PCQ indicators; for instance, the practice may access statistics about the share of diabetes patients with low HbA1c either by navigating to the diabetes subsection of PCQ or by generating the diabetes-related MR report. However, the MR reports do not include a comparison with other PCPs. Another difference is that the MR reports allow the user to set the time period, chose patient group etc., while the indicators in PCQ are standardized measures with numerators and denominators based on evidence-based guidelines, with only a few optional settings. The PCQ section thus represents an easier way to view practice performance.

## Methods

### Data collection

Data on utilization and measurements of quality indicators was obtained from Medrave Software. For each PCP, the data includes information about page views and the number of unique users accessing the application. The data on use was available from the first date each PCP connected to the application until September 30th 2021. Consequently, we have information about the use of MR reports and the number of unique users since 2012 for most PCPs in our study population, and information about the use of the PCQ section of MR since 2016–2020 (depending on when the PCP first viewed a page in the PCQ section).

The regional health authority provided background information on the PCPs, such as the number of listed patients (monthly data July 2012 - December 2020) and ownership. We also added data on responses from a recent survey of Swedish PCP managers’ views on leadership, quality improvement, and audit and feedback initiatives (see Additional file 2), which included 43 of the 122 PCPs in our study population (response rate = 35%), and PCP-level information from the 2021 wave of the National Patient Survey (www.patientenkat.se). The patient survey is administered by mail to a random sample of patients having visited a PCP during the autumn each year.

### Study population

Out of 200 PCPs in the region, 132 were passively enrolled (all public PCPs) or actively gave their consent (private PCPs) to participate in the study. We excluded ten PCPs that had been operating for less than one full year in January 2019 (when it became mandatory to provide data to the PCQ section of MR), leaving a study population of 122 PCPs (104 public and 18 private).

### Variables

#### Utilization pattern

“Use” was operationalized as the monthly number of page views in the application. Each time a user loads a page, it is counted as a page view. We were able to distinguish between use of the PCQ section and use of other MR reports. Additionally, we were able to study the use of diabetes-related pages in both the PCQ section and MR reports. Panel A, Table 1 shows the definitions of variables used to describe the utilization pattern.

**Table 1:** Variable definitions for PCQ and MR measures

**PANEL A**			
***General use***		***Use of diabetes sections***	
*PCQviews*_*it*_	Number of page views in the PCQ system by PCP *i* in month *t*. The month index *t* is relative to the first month a unit viewed a page, i.e., it does not refer to the same month for two PCPs that accessed the PCQ system for the first time at different points in time. Two versions: raw and per 1,000 patients.	*PCQ diab views*	Mean number of views in diabetes section of PCQ app, computed over Nov 2018-Feb 2020
*Mths in MR app*	Counting from September 2021, the number of months passed since the PCP first viewed a page in any part of the MR application.	MR reports diab views	Mean number of views of reports related to diabetes in MR app (excluding PCQ), Nov 2018-Feb 2020
*Mths in PCQ*	Counting from September 2021, the number of months passed since the PCP first viewed a page in the PCQ section of the MR application.		
*Share mths with PCQ view*	Share of months (between first use and Sep 2021) in which the PCP viewed at least one PCQ page (%).		
*MR app users*	Number of unique users that viewed at least one MR report; average calculated over all months since the PCP first connected to the MR app. Two versions - raw and per 1000 patients.		
*MR report views*	Number of reports viewed in the MR application; average calculated over all months since the PCP first connected to the MR app. Two versions - raw and per 1000 patients.		
**PANEL B**			
***Quality indicators (selected)***:		***Diabetes quality;*** share of diabetes patients with…:	
HbA1c_miss*	No record of HbA1c (share of diabetes patients)	*HbA1c_low*	…HbA1c < 52
Miss_albumin*	No record of albumin in urine (share of diabetes patients)	*BP_low*	…Blood pressure ≤ 140/85 mm/Hg
*Inf17Neg*	Antibiotic-treated pharyngotonsillitis with a negative near-patient Rapid Antigen Detection Test (RADT) (share of episodes last 12 months)	BP_miss*	…No record of blood pressure
*PPI_diag*	Patients with proton pump inhibitors who have an evidence-based indication (share)	*Statins*	…Prescribed statins
*CVD_risk*	Patients with co-morbidities who have been assessed for cardiovascular disease risk (share)	*Follow_up*	…Regular follow-up visit, any profession
		*No_albuminuria*	…No albumin in urine
**PANEL C**			
***Prevalence indicators (selected)***			
*Diabetes type 2*	Share of listed patients with a diabetes type 2 diagnosis (%)		
*Pharyngotonsillitis*	Number of patients with a pharyngotonsillitis diagnosis per 1,000 patients.		
*Multimorbidity*	Share of listed patients with at least two chronic conditions (%)		

Panel A shows definitions of variables used to describe the general use of the MR software (left) and the explanatory variables used in the regression analysis of the use of the diabetes section of the software (right). Panel B shows definitions of the studied quality indicators in the descriptive analysis (left) and the regression analysis for diabetes (right). Panel C shows definitions of the variables measuring prevalence of the conditions studied in the descriptive analysis. * Data may be missing for different reasons: either the measure was not taken at all, or it was not reported or documented in a readable way by the automatic extraction functionality. For HbA1c, missing data can only be due to the first reason (i.e., it is not due to deficient reporting or documentation). For the measures of albumin in urine and blood pressure, all three reasons are possible

We calculated two versions of the overall use variable: one raw and one standardized by the PCP size (views per 1,000 patients). Because we only had access to information about the number of listed patients until the end of 2020, we assumed that the number of patients remained the same from December 2020 through 2021 when calculating the measures per 1,000 patients.

#### Group indicator of high/low users

To compare PCPs with relatively high and low use of PCQ, we constructed a binary group indicator as follows:

1 $${PQviews}_{i} =\frac{\sum _{t=1}^{T}PQview{s}_{it}}{T}$$

For each PCP, this measure indicates the average number of monthly PCQ page views. We computed the average over all months $T$ between the first month when a PCP used PCQ and the last month in the data. Because larger PCPs may have more employees and thus users, we used the number of page views per 1,000 patients to construct the group indicator. Using the distribution of this variable, we divided the PCPs into two equally-sized groups: high user = above median, and low user = below median.

#### Primary care quality indicators

Since the main purpose of the PCQ library is to help QI at PCPs, most indicators are process measures indicating the adherence to treatment guidelines (prescriptions, follow-up visits), but there are also some indicators of intermediate outcomes (e.g., share of patients meeting blood pressure targets). For our comparison of high and low users, we selected a number of PCQ indicators that are relatively easy to interpret (leftmost part of Panel B, Table 1; see Additional file 1) together with relevant indicators of prevalence (Panel C, Table 1). We also studied PCQ indicators related to diabetes care and outcomes (rightmost part of Panel B, Table 1).

#### Background variables

We used the following variables from a regional *administrative system*: Number of listed patients, ownership type (public/private), morbidity of listed patients (a diagnosis-based risk score from the Johns Hopkins Adjusted Clinical Groups ® system (ACG), relative to the regional average; high value = high morbidity) and a social deprivation index of listed patients (average Care Need Index (CNI) weight; high value = high deprivation) [18, 19].

We used data on patients’ overall impression of the PCPs from the 2021 national patient survey (NPS). The overall impression variable ranges between 0 and 100 and is based on the proportion of positive answers (3–5 on the Likert scale) to certain questions in the survey (see [20] for details on questions and composition of scores).

We used responses to several questions from a *survey of PCP managers* conducted in the spring of 2022 (see Additional file 2). We computed binary variables equal to 1 if the respondent agreed to a large extent or fully (4 or 5 on a Likert scale) and 0 otherwise (1–3 on a Likert scale). We chose a cutoff of 4 instead of 3 because many managers answered 3, so many variables would not display any variation if we had grouped answer categories 3–5.

### Data analysis

#### Use of PCQ

In the analyses of utilization patterns, the unit of analysis was a PCP-month. To summarize how the use of PCQ varied since the PCPs first viewed a PCQ page, we computed means and medians of the monthly number of page views. To illustrate how the use changed with experience, we plotted the statistics against the number of months passed since the PCP first viewed a PCQ page.

#### Characteristics of high/low users

To characterize high users, we obtained descriptive statistics for the high and low users (see Eq. 1). We used t-tests to evaluate if the differences in means between the two groups were statistically significant. The variables in this analysis were either time-invariant or the first observed values.

Since manager survey data was not available for all PCPs, we performed a separate analysis of this data. For each survey question, we constructed a frequency table showing the number of responses by group (high vs. low users) and used chi2 tests to examine if there were statistically significant differences between the distributions.

#### Relationship between use and values of quality indicators

To examine if there was a relationship between the use of PCQ and the measurements of the PCQ indicators, we studied the changes in indicator values over time for eight diabetes related PCQ indicators (Panel B, Table 1). The unit of analysis was a PCP.

The indicator values change slowly over time as they are computed as rolling averages over information from the past 12–18 months. The changes were therefore computed as the difference between the value in March 2020 (i.e., before the Covid-19 pandemic) and the first month when all the diabetes indicators were available, i.e., Nov 2018 (the first month was Oct 2018 for all except one indicator).

We estimated the following linear regression specification of the changes in indicator values:

$$\begin{array}{l}\Delta indicator\, = \,\alpha \, + \,{\beta _1}\,*\,PCQ\,diab\,views\, + \\\,{\beta _2}\,*\,MR\,diab\,views\, + \,{\beta _3}\,*\,Indicato{r_{2018}}\, + \,\epsilon \end{array}$$

The main independent variable of interest, $PCQ diab views$, counted the number of views of pages containing diabetes-related PCQ indicators, measured as the mean over Nov 2018-Feb 2020. We also controlled for the number of views of diabetes-related MR reports ($MR views$). MR diabetes report includes many indicators that are also available as PCQ indicators (i.e., the HbA1c distribution, blood pressure distribution, patients with no information on albuminuria) but not all (i.e., follow-up visits), and it does not provide comparisons with other practices. In a sensitivity analysis, we omitted the control for the number of MR diabetes reports.

All models were adjusted for the initial (i.e., Nov 2018) value of the indicator ($Indicato{r}_{2018}$). The models were estimated in Stata 16.1 using heteroscedasticity robust standard errors.

## Results

### First use of PCQ

Eight PCPs used PCQ for the first time during the pilot project that started in 2016. Nine other PCPs used PCQ for the first time during 2017, and 15 in the first half of 2018. The bulk of first use occurred around the time when it became mandatory to enable the automatic extraction functionality; 58 PCPs premiered in the second half of 2018, and 28 in the first half of 2019. The last four PCPs used PCQ for the first time in the second half of 2019 or in January 2020.

The rapid increase of first use just around the time when it became mandatory to enable automatic data extraction was highly concentrated to public PCPs. Among the private units, the timing of the first use was more evenly spread out, ranging from the pilot phase to January 2020.

At the end of our observation period, all PCPs in our study population had used PCQ for at least 20 months (mean = 37, median = 34). Consequently, we were able study PCQ use for almost two years for most of the PCPs. The PCPs’ use of the MR application (i.e., MR reports) dated even longer: at the end of the study period, the PCP with the shortest experience of MR used the application for the first time 52 months earlier (mean = 102 months, median = 107 months).

### Use patterns of PCQ

**Fig. 1:**
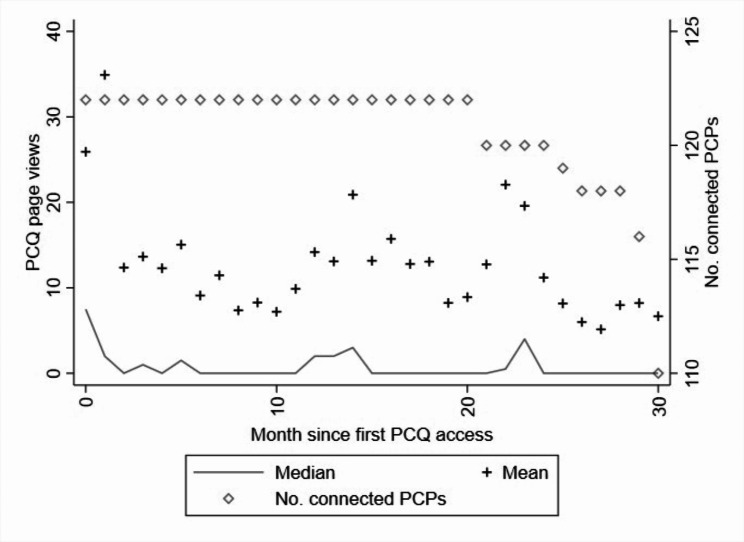
Monthly number of PCQ page views, by time since first page view The horizontal axis shows the number of months passed since a PCP viewed a PCQ page for the first time (censored at 30 months). The rhomboids indicate the number of PCPs connected to PCQ for a given number of months (right vertical axis). Descriptive statistics for the number of PCQ page views per month since first connection (mean/median) can be read off the left vertical axis

Figure 1 shows the development of PCQ use, measured as the monthly number of PCQ page views, from the month a PCP first viewed a page in the PCQ section of the application and onwards. For each month, the mean (+) and median (solid line) number of page views can be read off the left y-axis. For each month, the rhomboid indicates the number of PCPs in the study population that were observed as a user for at least this long (right y-axis). The rhomboids are included to highlight that not all PCPs used the PCQ section for as many as 30 months, and that the statistics for month 20 onwards thus are based on a subset of the total population.

As shown in Fig. 1, the number of page views tends to be the highest just after a PCP first accesses the PCQ section of the MR application. The mean number of PCQ page views in the first month is almost 30 and the median is almost 10 views. The activity drops sharply after the first two months, with around 10 views per month on average. The line representing the median shows that in a given month, fewer than half of PCPs look at any page in the PCQ section of the MR application. Yet, 75% of the PCPs used PCQ at least one third of the months (e.g., for a PCP connected to PCQ for 20 months, this corresponds to slightly more than 6 months), and 90% used it at least 25% of the months. The four PCPs with the lowest frequency of use had viewed a PCQ page in 17–21% of all months since their first access.

In spite of the drop in activity after the first months, Fig. 1 indicates a somewhat periodic pattern, suggesting increased activity levels around one year after the first connection, and then again almost 2 years after.

We also constructed a similar graph for a standardized measure; the number of page views per 1,000 listed patients. The pattern over time was very similar. In the first two months, the mean number of page views per 1,000 listed patients was around 3.5. In the subsequent months, the mean fluctuated around 1.5 views per 1,000 patients.

The mean and median number of unique users logging in to any part of the data visualization application was four (interquartile range 3.36–4.69). Thus, it was common to have a handful of unique users at each PCP each month.

### Characteristics of high users

Table 2 shows descriptive statistics for the high and low users of PCQ (defined by Eq. 1), together with *p*-values of t-tests of the null hypothesis of no difference in means between the groups.

**Table 2:** Descriptive statistics for high and low users of PCQ

	Low user (n = 61)					High user (n = 61)				
	Mean	SD	Min	Max		Mean	SD	Min	Max	p-val
No. patients	9,891	3,956	29,545	19,210		8,139	2,956	2,852	14,757	0.006
Morbidity (ACG)	1.03	0.12	0.79	1.29		1.03	0.12	0.82	1.39	0.907
Socioeconomic deprivation (CNI)	2.3	0.7	1.52	4.5		2.28	0.77	1.38	5.29	0.903
Private	0.15	0.36	0	1		0.15	0.36	0	1	1
**MR application use**										
Mths in MR app	100.7	12.3	52	108		102.41	10.94	56	111	0.42
Mths in PCQ	36.75	8.63	20	66		36.25	7.87	20	63	0.735
MR app users	3.89	1.16	0.95	6.94		4.11	1.14	1.05	6.32	0.299
MR app users/1000	0.44	0.16	0.11	0.99		0.56	0.23	0.25	1.49	< 0.001
MR report views	62.88	29.54	16.15	167.07		68.67	30.98	13.53	175.43	0.293
MR report views/1000	6.65	2.53	1.82	14.8		8.96	4.2	3.01	26.23	< 0.001
**PCQ use**										
PCQ views	6.66	4.28	0.67	19.65		17.16	9.17	3.92	49.89	< 0.001
PCQ views/1000	0.65	0.29	0.11	1.1		2.29	1.36	1.1	7.7	< 0.001
Share mths w. PCQ view	41.17	13.9	17.24	79.41		56.56	16.3	22.86	95.83	< 0.001
**PCQ and prevalence**										
Diabetes type 2	0.05	0.01	0	0.07		0.05	0.01	0.03	0.09	0.837
- HbA1c_miss	0.08	0.03	0	0.15		0.08	0.03	0	0.17	0.868
- Miss_albumin	0.21	0.12	0.08	1		0.19	0.08	0.06	0.52	0.355
Pharyngotonsillitis	32.2	21.89	9.85	162.79		30.97	12.23	13.14	63.21	0.701
- Inf17Neg	0.11	0.05	0.02	0.24		0.11	0.06	0	0.25	0.345
Multimorbidity	0.2	0.05	0.01	0.29		0.21	0.04	0.13	0.3	0.746
- CVD_risk	0.7	0.1	0.25	1		0.7	0.1	0.22	0.87	0.83
- PPI_diag	0.39	0.1	0	0.66		0.42	0.1	0.15	0.63	0.058
Patient satisfaction	78.18	6.64	61.7	91.7		79.86	7.17	60.2	94	0.181

Descriptive statistics by type of user, low or high. Low (high) users are defined by the within-PCP averages of the monthly number of PCQ page views (over the whole observation period): low (high) user = below (above) the median for all PCPs (see Eq. 1). The *p*-values come from t-tests of equality of means between the groups. For variable definitions, see main text and Table 1. The first part of the table describes general background characteristics. The second and third parts describes the use of the MR and the PCQ applications; in both cases the variables counting months (mths) count backwards from September 2021. The fourth part shows indicator values and relevant prevalence rates for selected PCQ indicators; the values are from the first month each indicator was observed (Feb 2019 for Lm1 and Oct 2018 for the other indicators). The last row shows the overall patient satisfaction rating according to the 2021 survey wave. All other variables are means over the study period

The high use PCPs were smaller (mean = 8,139 vs. 9,891 patients), but were similar to low use PCPs in terms of morbidity, social deprivation, and ownership type. The two groups of PCPs had similar lengths of experience of the PCQ section as well as the rest of the data visualization application (around 36 and 100 months, respectively), and a similar number of unique users. However, the number of users per 1,000 patients was larger in the high-use group, probably due to their smaller size. Further, the number of MR report views were similar on the total, but the number of reports per 1,000 patients was almost 50% higher in the high use group (8.96 vs. 6.65).

Since we used the average number of PCQ views (per 1,000 patients) over the whole period to define the two groups, it follows automatically that the mean number of PCQ page views differed substantially between the groups; the difference was statistically significant. The statistics for the minima and maxima show that the largest average number of PCQ views per 1,000 patients was 1.10 in the low use group, implying that the same number was the smallest average number in the high use group. In the latter group, the maximum average number of page views per 1,000 patients was 7.70.

Yet, within each group, there was substantial variation in use. Also, some PCPs who were classified as low users accessed the PCQ section of the application often, but then only viewed a small number of pages.

The lowest part of Table 2 shows that high and low users had similar prevalence of patients with diabetes, pharyngotonsillitis, or multimorbidity, and they performed similarly on the selected PCQ quality indicators. The exception was the indicator for the share of patients with a PPI prescription whose EMR included information on an etiologic diagnosis (as an indication for the treatment), which was slightly higher in the high-user group (*p* = 0.058).

With regards to the five quality indicators, it is worth noting that the standard deviations were quite large, indicating substantial variation between the PCPs in terms of performance. For instance, the share of patients with multimorbidity whose risk for cardiac problems had been assessed ranged from 22 to 100% (mean 70%, sd 10% in both groups). Similarly, the share of type 2 diabetes patients with no information on albuminuria ranged from 6 to 100% (mean 20%, sd 8–12%). Notably, these measures were taken before the covid-19 pandemic.

### Manager survey

We also compared the two groups with respect to the responses of the manager survey (Additional file 2). Notably, there were only 43 respondents from our study region (22 low-users and 21 high-users).

The share of managers with a nurse education and the share engaged in clinical work (part-time managers) was similar in the two groups. The share of respondents considering the GP staffing level better or much better than the average was larger in the group of low users (11/21 vs. 6/20), but the difference was not statistically significant.

A slightly higher share of respondents from high-user PCPs stated that their leadership was highly focused on *ensuring professional norms and quality in the clinical work* (20/21 vs. 17/22; *p* = 0.089). However, there were no differences in the distributions of related questions such as their focus on *developing and implementing new routines and processes, ensuring adherence to guidelines*, or continuous work to *align routines and processes to meet medical needs*.

The managers in the high use group were more likely to fully or to large extent agree with the statement that *clinical professionals in the PCP take own initiatives to make changes in response to perceived needs* (16/21 vs. 7/20, *p* = 0.008). However, the two groups did not differ in their view on the *motivation to adopt new ideas and solutions to existing problems*, or in the *availability of routines for implementing novel solutions*.

There were no apparent differences between the two groups with respect to how they viewed the audit and feedback from the governing regional health authority.

### Association between use and diabetes indicator values

The regression results in Table 3 show that the number of page views in the diabetes section of the PCQ application was statistically significantly associated with changes in two of the eight diabetes indicators. First, the number of diabetes related PCQ page views was associated with a reduction in the share of patients with no recorded HbA1c. A one standard deviation increase in the number of PCQ diabetes page views was associated with a 0.3% point decrease in the proportion of patients with no recorded HbA1c information; a decrease of 4% in relation to the mean proportion of 7.7% patient with no recorded information. Furthermore, an increase in the number of PCQ diabetes page views of the same size was associated with a 0.8% point increase in the share of patients with blood pressure ≤ 140/85 mm/Hg; an increase of 1.2% in relation to the mean proportion of 64.4%.

**Table 3:** Regression results for change in diabetes indicators

	(1)	(2)	(3)	(4)
	**HbA1C_low**	**HbA1C_miss**	**BP_low**	**BP_miss**
PCQ diab views	-0.002	-0.003	0.008	-0.002
	[-0.009,0.005]	[-0.006,-0.000]	[0.001,0.014]	[-0.007,0.003]
MR reports diab views	-0.003	-0.001	0.001	-0.001
	[-0.011,0.004]	[-0.005,0.002]	[-0.008,0.010]	[-0.006,0.003]
Indicator_2018_	-0.515	-0.280	-0.316	-0.324
	[-0.793,-0.236]	[-0.446,-0.114]	[-0.649,0.017]	[-0.557,-0.092]
Constant	0.282	0.027	0.182	0.032
	[0.130,0.435]	[0.012,0.042]	[-0.025,0.389]	[0.013,0.050]
N	122	122	122	122
R2	0.513	0.161	0.159	0.129
Mean Indicator_2018_	0.526	0.077	0.644	0.075
	(5)	(6)	(7)	(8)
	**Statins**	**Follow_up**	**No_albuminuria**	**Miss_albumin**
PCQ diab views	-0.003	0.001	-0.003	0.004
	[-0.008,0.002]	[-0.003,0.004]	[-0.009,0.004]	[-0.002,0.011]
MR reports diab views	0.008	0.000	-0.008	0.006
	[0.001,0.014]	[-0.003,0.004]	[-0.015,0.000]	[-0.003,0.015]
Indicator_2018_	-0.356	-0.343	-0.423	-0.427
	[-0.673,-0.038]	[-0.526,-0.160]	[-0.701,-0.144]	[-0.700,-0.154]
Constant	0.227	0.314	0.088	0.262
	[0.038,0.416]	[0.143,0.486]	[0.035,0.140]	[0.089,0.435]
N	122	122	122	122
R2	0.284	0.142	0.368	0.330
Mean Indicator_2018_	0.582	0.933	0.202	0.608

Coefficient estimates from eight linear regressions. In each model, we regressed the change in a PCQ indicator between November 2018 and March 2020 (the dependent variable) on the mean number of page views in the diabetes section of PCQ or MR over the same period (independent variables) and the initial level of the indicator. The PCQ and MR variables are standardized, i.e., the coefficients can be interpreted as the marginal effect of a one standard increase in the variable. One standard deviation of *PCQ diab views* = 0.787 views/month. One standard devation of *MR reports diab views* = 4.55 views/month. The rows named “**Indicator**_**2018**_” show the marginal effects of an increase in the initial level of the indicator. The mean of the initial indicator levels are also displayed in the table (rows named “**Mean Indicator**_**2018**_”). The unit of analysis is a primary care practice. 95% confidence intervals in brackets. See Table 1 for variable definitions

Use of diabetes-related MR reports was statistically significantly associated with one of the eight indicators, the share of patients on statins. A standard deviation increase in the number of report views was associated with an increase of 1.4% of the mean (58.2%).

The number of diabetes-related PCQ page views were positively correlated with the number of diabetes-related MR reports (standardized regression coefficient of 0.18 in a bivariate regression, *p* = 0.046). Yet, the results from the sensitivity analyses in which we omitted the MR reports variable were similar.

## Discussion

The user statistics analyzed in this study shows that almost all studied PCPs accessed the PCQ measurements now and then. However, less than half of all providers looked at any PCQ indicator in any given month, and the low average number of pages views indicates that PCPs usually explored a small subset of all available indicators. After an initial period of two months, the number of page views decreased markedly. This pattern may reflect that PCPs took part in educational activities offered by the region in the first months, but did not manage to build up their own routines to use the system. It is also possible that the curiosity waned after the initial period, or that users learn to navigate the application after a while (thus finding their way with fewer clicks) or decided to focus on certain areas for QI.

A limited use of PCQ is consistent with findings of earlier studies focusing diffusion of health care innovations. Many promising innovations in health are characterized by slow adoption or even non adoption, in spite of attempts to scale up and support diffusion [21, 22]. Similar to other PC contexts [23], recent qualitative studies of existing audit and feedback practices in Swedish PC suggest that professionals and PCP managers do not experience the environment as supportive of QI work [16]. Lack of time for QI, lack of autonomy and lack of QI initiatives at health system levels are considered as barriers to their own QI. Moreover, existing audit and feedback practices are perceived as external and coercive, focusing fulfillment of contracts with payers, with limited support of bottom-up driven and more complex change [17].

Overall, we did not find substantial differences between PCPs categorized as high or low users. Both groups were similar in terms of patient characteristics (morbidity, social deprivation, patient satisfaction), had used the data visualization application for similar lengths of time, and performed similarly on selected quality indicators at baseline. However, high users of PCQ also tended to view more MR reports. Moreover, managers of such PCPs were more likely to agree with the statement that the staff take own QI initiatives. These two results suggests that the use of PCQ hinges on the motivation of individual users – both managers and staff. This is in line with the intention of the PCQ initiative, which aims at supporting QI work “bottom-up”. The commitment and motivation of health professionals is crucial for organizational change [24, 25]. This also implies that differences in motivation, may lead to systematic quality differences between PCPs, if the use actually leads to quality improvements.

The analysis of the PCP’s use of pages related to diabetes showed a few statistically significant associations between the number of page views and the changes in quality indicators over an almost 18 months long period. Out of 16 estimates, three were statistically significant at the 95% level, more than would be expected by chance. Two of these estimates related to the use of the diabetes pages in the PCQ section and one to the use of diabetes-related MR reports. This suggest that the opportunity to compare one’s own performance with the performance of other PCPs (an opportunity only available in the PCQ section) affects behavior in a complementary way to information on the performance of one’s own PCP only (supplied in the MR reports). The simplicity of PCQ, which comprises a ready-made selection of evidence-based indicators with no need to make special settings, could also contribute to this effect.

Two of the statistically significant indicators were process measures: the share of patients with current HbA1c result and the share who has a prescription for statins. Since all laboratory tests and prescriptions are automatically documented in EMRs (as well as extracted automatically by the MR application) they cannot be improved by better “reporting” or documentation. On the contrary, to improve these measures the missing patients have to be identified, contacted and probably invited for a visit for prescription and a blood sampling. Possibly, the routines for regular check-ups of diabetes patients in general also must be improved. The third statistically significant association related to an intermediate outcome, the fraction with normal blood pressure. This result could be explained by better documentation of blood pressure or, of course, by better treatment and real improvement of blood pressures. It seems reasonable to assume that both blood pressure and HbA1c was addressed when seeing the patients. Although the association between the absence of current blood pressure values (BP_miss) and PCQ use was not statistically significant, the estimate was indeed negative.

The positive associations between use and changes in performance, together with substantial variation in the use of the PCQ section of the application, open up for questions regarding how regional health authorities can stimulate an increased use, while still retaining the professional-led spirit of PCQ. Even if data is accessed and enhances health professionals’ intention to improve quality, an intention-to-action gap may arise if health professionals misinterpret the data or struggle to identify actions that could lead to QI [26].

Increased interest and improved capabilities to use the data may be strengthened by social interaction with peers. Previous studies on audit and feedback suggests that understanding of the data and commitment to action planning and change can be facilitated by social interaction with peers in a group setting [26, 27]. A previous Swedish study reports that PCPs who engaged in peer discussions on data and guidelines indeed displayed better adherence to antibiotics treatment guidelines [28]. Collegial feedback is far from a new idea. Such practices have existed for decades in Swedish PC [29] and collegial discussions are becoming more common in many European countries [30, 31]. However, systematic support from regulators and payers is often lacking. Automated access to valid and up-to-date data is improving fast due to digital technology. Without additional support and opportunities for change, the use of data for QI and implementation of change is likely to be uneven at best.

### Strengths and limitations

The main strength of this study was the access to unique longitudinal data on the use of PCQ across a substantial number of practices in a large Swedish region, linked to multiple sources of information about practice characteristics.

A notable feature of the study setting was that, beyond the requirement to enable the automatic data extraction from EMRs, users were not commanded “from above” to use the application. Thus, the setting made it possible to learn about user-driven utilization of an application visualizing data on performance indicators.

The study had a number of limitations. The number of page views is a coarse measure of the extent to which managers and staff meaningfully engage with the content in the PCQ section of the application. For individual users, ten page views per month might reflect a mindful monitoring of carefully selected indicators, as well as a mindless procrastination exercise. However, the fact that the three statistically significant associations between use and indicators all pointed in the direction of improvements (i.e., fewer missing HbA1C, lower blood pressure, more prescribing according to recommendations) give some support for the former interpretation when it comes overall use at the group level. At the same time, there are notable limitations of how these associations can be interpreted. The observational study methodology does not allow for causal interpretations. For instance, practices also use the application to report data to the national diabetes quality register. Practices that for other reasons had decided to improve their reporting to the quality register may have started to explore the diabetes pages in the application once they had logged in to submit data. It is also possible that clinicians with a special interest in diabetes are both particularly likely to look at the diabetes indicators, and to make other efforts that affect performance. Furthermore, changes in measures such as the share of patients with up-to-date measures of blood sugar or acceptable blood pressure may reflect efforts on part of patients, on part or the care system – including changes in documentation in EMRs, and pure chance. Thus, the indicators are imperfect measures of primary care quality. Another limitation of our study was that the data did not indicate which quality indicators that caught the most interest among users. Using more granular data to study this question is a promising area for future research.

For feasibility reasons, we had to limit our analysis of the associations between use and performance to a subset of quality indicators in the PCQ section of the application. We chose diabetes, which is an important area in primary care. It is possible that the associations were attenuated since the PCPs could access some of the indicators (though not comparisons with other practices) in other parts of the data visualization application even before our study period.

## Conclusion

This study report findings from a study of a new initiative in Swedish primary care, the PCQ, intended to support QI initiatives led by professionals. Most PCPs in our study showed interest in the automated feedback reports with comparative information on performance indicators, although the intensity of use varied and was rather limited in general. Fewer than half of all PCPs looked at any PCQ indicator in a given month, although most accessed the PCQ data at least once every third month. PCPs typically viewed information about only a small subset of all available quality indicators in PCQ. PCPs that viewed many indicators related to diabetes showed relatively large improvements in diabetes care. The positive associations between use and changes in performance suggest that policymakers should increase their support of PCPs QI work. To support QI work, while still retaining the profession-led philosophy of the PCQ initiative, support of collegial feedback interventions should be considered.

While this study was set in a Swedish context, it provides inspiration and policy guidance reaching outside the study setting by showing that a profession-led initiative with easily accessible and clinically relevant data may spike interest in quality improvement work. Further, the need for external support to stimulate continuous use of the data may be relevant also in other contexts.

### Electronic supplementary material

Below is the link to the electronic supplementary material.

**Additional file 1:** The Swedish Primary Care quality Initiative

**Additional file 2:** The survey to practice managers

## Data Availability

The datasets analysed during the current study are available from the corresponding author on reasonable request.

## List of abbreviations

A&F: Audit and feedback
ACG: Adjusted Clinical Groups
CNI: Care Need Index
COPD: Chronic Obstructive Pulmonary Disease
CVD: Cardiovascular Disease
EMR: Electronic medical records
GP: General practitioner
MR: Medrave
PC: Primary care
PCP: Primary Care Practice
PCQ: Primary Care Quality (“Primärvårdskvalitet”)
PPI: Proton Pump Inhibitor
QI: Quality improvement
