# Supplementary material for: Access to automated comparative feedback reports in primary care – a study of intensity of use and relationship with clinical performance among Swedish primary care practices
Source: BMC Health Serv Res. 2024 Jan 4;24:33. doi: 10.1186/s12913-023-10407-9 (PMC10768433; doi:10.1186/s12913-023-10407-9)
Supplement: Supplementary file 1 — Additional file 1: The Swedish Primary Care quality Initiative [file 12913_2023_10407_MOESM1_ESM.docx]

# Additional file 1: The Swedish Primary Care Quality Initiative

## Indicators

The indicators in the Primary Care Quality library cover different chronic diseases, common infections and non-disease-specific measurements, such as continuity of care, prioritisation of patients with high needs, and comorbidity, see table below. Each topic (e.g. diabetes, heart failure, or pneumonia) consists of around 6-10 different indicators. For all included chronic diseases, there is an indicator for the share of patients with the diagnosis, and one indicator measuring the share of patients who have had check-ups during a certain time frame. Additional indicators in the chronic disease group reflect certain diagnostic procedures, monitoring of the disease, treatment (e.g. drug treatment) and physiotherapeutic interventions. The indicators on infectious diseases mainly capture the use of antibiotics. The non-disease specific measures include indicators that reflect the broad mission of primary care, and measures that don’t depend on a specific disease.

| **Chronic diseases** | **Infectious diseases** | **Non disease-specific measures** |
| --- | --- | --- |
| Anxiety  Asthma  Atrial fibrillation  COPD  Coronary artery disease  Dementia  Depression  Diabetes  Heart failure  Hypertension  Kidney disease  Leg ulcers  O[steoarthritis](https://www.google.com/search?sxsrf=ALiCzsaa4gENz8yNYSNBnL-X04PJ71ZWNw:1663181272826&q=Osteoarthritis&spell=1&sa=X&ved=2ahUKEwjgxdqH-ZT6AhXJpIsKHa9EDR4QkeECKAB6BAgBEDs)  Osteoporosis  Stress-related problems  Urinary incontinence | Respiratory tract infections  Urinary tract infections  Skin infections  Covid-19 | Comorbidity  Cooperation with other health care  Drug treatment  Elderly  Lifestyle habits  Prioritization  Sick leave  Sustainability (environment) |

## Interpreting the indicators

For many indicators, it is difficult to give an exact target value or sometimes even say whether it is better to have a high or low value. It depends on various factors such as the patients' other diseases and age profile. Moreover, prevalence of chronic diseases and lifestyle habits often reflects socio-economic conditions in populations rather than performance of the primary care practice. Indicators also depend on each other. E.g., a low prevalence of a chronic disease may indicate a healthy population or that the disease has not been detected. If only few of the patents with a certain disease are identified it is possible that a high share of them should have a certain treatment. Conversely, a high incidence may indicate an unhealthy patient population, but it may also indicate over-diagnosis. If the latter is the case, only a low percentage of them may need the treatment. The automatic data extraction from EMRs has the advantage that the data is always up to date and that no extra administration is needed for data registration. However, it also makes data quality dependent on what is routinely noted in the EMR.

For this study, we selected measurements which are not vulnerable to errors due to the automatic data extraction, and which are relatively easy to interpret. Two of the measures reflect prevalence on chronic disease and are used to compare the morbidity in the populations at the different primary care practices. Three of the measures reflect almost indisputable quality: Regular check of albuminuria and blood glucose (HbA1C) for patients with diabetes is recommended in most guidelines, and so is avoiding antibiotics to patients with negative rapid strep test.

Examples of measures that were deselected, since interpretations is not always straight forward, include:

| Proportion of patients with asthma and maintenance treatment who had a return visit (As1A). | All professions - all patients do not need a return visit. |
| --- | --- |
| Proportion of patients with leg ulcers who have an etiological diagnosis (Be2 Leg ulcers BE02). | Few patients. Nurses often manage these patients and they rarely use code diagnoses which can lead to incorrect values. |
| Proportion of patients with diabetes who have blood pressure ≤140/85 mmHg (Di3L1-4). | Many blood pressure values are not included in the output (home blood pressure and ambulant measuring). |
