# Supplementary material for: Access to automated comparative feedback reports in primary care – a study of intensity of use and relationship with clinical performance among Swedish primary care practices
Source: BMC Health Serv Res. 2024 Jan 4;24:33. doi: 10.1186/s12913-023-10407-9 (PMC10768433; doi:10.1186/s12913-023-10407-9)
Supplement: Supplementary file 2 — Additional file 2: The survey to practice managers [file 12913_2023_10407_MOESM2_ESM.docx]

# Additional file 2 – Survey to practice managers´

Data about practice managers’ background and managers perceptions about their leadership style, the approach to audit and feedback from the region and innovation climate at the primary care practice was collected through a web-survey. Besides background questions about the respondent and the primary care practice, the survey contained nine sections of Likert-scale questions and statements. The survey was distributed as a link via e-mail to managers of primary care practices in February 2022. Two reminders, in the form of a letter with the questionnaire attached, were sent out. The first reminder was sent out four weeks following the e-mail and the second reminder two weeks later. The target population for the survey was managers of all 1 327 primary care practices in Sweden. In this study, responses from managers in the study population (n=122) and results from questions in three sections was used (see table below, direct translation from original Swedish language). The response rate (in total 35%, 43 respondents) was not statistically significantly different for low users (36%, 22 respondents) compared to high users (34%, 21 respondents).

| **Managers’ background and staffing situation at the primary care practice** | **Anchors/ alternatives** |
| --- | --- |
| What is your professional background?  If other, please specify  In your current position, do you share your working time between administrative and clinical work?  If yes, to what extent to you perform clinical work?  If no, do you have other assignments outside the primary care practice?  What type of assignments?  How many years of experience do you have at your current position?  How many years of experience as a manager in primary care in total do you have? | GP/RN/Other  Free text  Yes/No  0-100%  Yes/No  Free text  No of years  No of years |
|  |  |
| Staffing situation |  |
| How is your staffing situation in regards to GPs in comparison with other primary care practices in the region? | 1=much worse  5=much better |
|  |  |
| **Approach to Audit & Feedback (A&F) from the regional health care authority** |  |
| The A&F from the region as a purchaser of services…   - Focuses on support and feedback to the primary care practice to achieve goals related to clinical quality, patient satisfaction, continuity and access. - Focuses on adherence to guidelines and requirements in agreements with the region. - Provides us with information about deviations from guidelines and targets and our performance relative to other primary care practices in the region. - Facilitates quality improvement work through improved knowledge of other primary care practices and best practices. - Data used in A&F from the region is verifiable and updated. - Data used in A&F from the region is sensitive, i.e., it captures changes in quality at the primary care practice. | 1 = Fully disagree  5 = Fully agree |
|  |  |
| **Managers’ leadership style** |  |
| In my leadership and management of the primary care practice, I focus on…   - Ensuring professional standards and norms in clinical work. - Developing and implementing new routines and working methods. - Ensuring adherence to existing guidelines and routines. - Continuously improving working methods and routines to meet the needs and preferences of our patients. | 1 = Fully disagree  5 = Fully agree |
|  |  |
| **Innovation climate** |  |
| To what extent…   - Is there an interest in implementing ideas and solutions to problems identified by employees? - Do employees initiate change based on their own assessment of problems and needs at the primary care practice? - Do you encourage employees to engage in change behaviour and improvement work? - Are there structures and routines in place to implement suggestions for change and solutions to problems identified by employees? | 1=Very low extent  5= Very high extent |
